# Letter to the Editor

**Published:** 1870-05

**Authors:** 

**Affiliations:** Chicago


### Epistolary.

My Dear Professor: It is a rimy, juicy day abroad, the east wind bearing the chill of death on its wings, piercing one to the very marrow. We have the "blues" terribly, and as is our custom, fall back upon "Burton's Anatomy of Melancholy," or the "Sorrows and Trials of a Nice Young Man," published somewhere about the middle of the 15th century; old, 'tis true, but replete with much consoling philosophy.

It is with some regret we say, our time has been so occupied with the harrowing cares and duties of life, as to have made it impossible to fill out our "skeleton records" to a proper and suitable article for your valuable Journal. What with fighting duns,—cajoling the determined, and bullying the weak;—staving off executions; pursuing refractory debtors; and "greasing the ways" of those ill in body and mind, for their passage over the "Stygian waters," we have been kept in a perpetual ferment, and have not had that placidity of mind, nor evenness of temper, consistent with connected and coherent thought; so thus the busy hours have sped, sweeping rapidly down the echoing corridors of Time, with saddening, and, may we not hope chastening, influences.

Events, whether professional or otherwise, are constantly transpiring in the life of every one, which, if properly appreciated and interpreted in a true philosophical spirit, must inure to the advantage of society at large; so, for want of better material, we have concluded to give you a few sketches of our wanderings—Asmodeus that we are—since last grasping your brotherly hand.

You may not yet have forgotten the Tipperary man, who with rage in his heart, and whisky in his head, dived down a steep stairway one night, fracturing his skull, besides receiving other contusions, and minor injuries! The flow of blood continued from the external meatus of the right ear, (more or less) for the first week; pulse 55; coma, with stertorous breathing; retention of urine; and involuntary fœcal discharge; partial anesthesia, and palsy of left side; and irregular sluggish pupils—the left much dilated—for at least three weeks longer, and then a gradual lighting up of the ugly symptoms, followed by some febrile reaction, and noisy, night delirium. Well, under constant nursing, and some judicious handling, he slowly recovered, and now seems possessed of a brighter intelligence than ever before. As he has not been of any account to his family for many years, it is questionable whether any good could accrue to them or society, by allowing him to live, unless, indeed, this severe shock to his cerebral mass may have churned up his dormant ideas to a fitter realization of the duties of life. We have heard of idiots and imbeciles dropping from the upper stories of houses, and losing several ounces of brain matter on the pavement below, thus rendering them more sensible and rational beings. And, *en parenthesis*, may we not say *amigo mio*, that a like process of smashing and extrusion would be of vast benefit to many of our acquaintances?

Again, we were taken by Dr. E. W. Edwards, not long since, to see a natural curiosity, a woman with that perverted condition of the nervous system, known as catalepsy—a religious enthusiasm, the prevailing element—who would not pass her water, and who would not, though she could, talk. Now, just observe this, a "bird who could sing, but wouldn't;" no persuasion of friends, no marital endearments, no entreaties of her physician, were of least avail; her tongue she would hold, and has done it to this very day. Wonderful freak of nature! and there she lay, or sat in her bed, those large melancholy eyes following our every movement, comprehending every expression, but not one word; reticent and determined as a female alone can be, when determined to balk her tormentors. Had she the physical strength, we should have suggested taking her to the museum, or exhibiting her to those windy creatures, with "luminous foreheads," the "Shrieking Sisterhood," as a model *she*, and totally exempt from that prevailing epidemic—the *cacoethes loquendi*—which being freely translated, means a "diarrhœa of speech." We also hinted to our wife to come down and take a look, but she indignantly refused our modest request, stating that she would talk, and liked to talk, and this was one of the prerogatives of her sex, and, bye the bye, this is as near as she ever got to women's rights, and we don't believe she will ever get nearer, if there is any virtue left in leather and sweet oil. Our patient is, however, recovering her health and strength, but still holds her peace; let her pass: and long may she wave—(*not* her tongue).

A few days ago we were requested to appear before one of the courts, to testify, as an expert, in the case of the People *vs.* Mary Smith, who several months back, in a fit of the frolics, popped away three or more barrels of her revolver at her brother-in-law, thereby doing him severe bodily injury, and endangering his life. As the law does not allow even such frisky females as Mrs. Smith to play with edged tools, she was arraigned by the people, and the plea of insanity, or, to draw it milder, mental derangement from chronic ear disease, was offered in her defense. Drs. E. S. Holmes, and H. R. Smith, both extensively acquainted with aural diseases, were also called to give their opinions, and the former gentleman, on examination of the lady's ears, states the following:

"*Right Ear.*—Tympanal membrane, slightly concave; bright spot, scarcely visible; external meatus, dry; and eustachian tube pervious after two expirations. *Left Ear.*—Same condition, except bright spot more visible; eustachian tube also pervious after two expirations."

Here we have an enumeration of symptoms belonging to many diseases of the ear, but sufficiently significant to frame something of a diagnosis; the sequel of some acute affection, several years past, perpetuated in a chronic form, by a strumous diathesis, or or some other condition of depreciated vitality, attacking not only the external ear, but also the middle, and yet, as her hearing was well preserved, creating no very great pathological changes.

The Judge talked very learnedly upon ear affections, and especially upon the terrors of ear-ache—and after a little confabulation among the lawyers, the plea was allowed; the Judge gave his opinion *ex cathedra;* the doctors drew in their horns, (we were not on hand); and Mrs. Smith was again let loose, to try her hand and her pistol upon her brother-in-law, the next time with perhaps better success.

We desire to say here, that the opinion was a bad one, and no medical man with the light before him, could predicate such, even upon the doctrine of remote sympathies. We grant that ear-ache is a terrible infliction, and know that a woman, with any severe pain, is very apt to become hysterical, cataleptic, lunatic, or any other thing you may suggest, and to crack away with all kinds of implements at the monster man; for example, *vide* any maimed man, with abridged rights — not ourselves, be it understood — but we mildly reject the conclusions of the learned lawyers, of whom we are none — *te Deum laudamus* — but retain our own from a knowledge of the disease, being doctors — *de profundis clamamus.*

We have lately been relieving the monotony of our lives by perusing the proceedings of the Chicago Medical Society, as published in the *Daily Times.* We have been favorably impressed with their debates; evincing great profundity of learning, extensive pathological research, and much public modesty of demeanor. We think they will compare with the learned meetings of the London and Parisian Societies, not that they generally take so extensive a range, but are very pithy and sententious. And now, while speaking of this Society, let me ask if they did not revise the last "tariff of prices"— a very necessary bill, to govern our charges for attendance upon the ill? We believe we are right, and acknowledge it a very laudable thing; but in our experience, and that not very long since, we discovered that the very framers of that bill, and those most persistent in recommending still higher fees, were the very physicians to under-bid, and would even go long distances for twenty-five or fifty cents, the more especially if they could oust a brother practitioner. And, *apropos* of this, have we not also a "Code of Ethics" under their jealous guardianship? but of what avail? Can there be shown a handful of men in our profession who care one copper for its generous rules, or who would observe a single principle of its laws, if by ignoring them, and injuring one another, they could make a point? No sir! it is the way of the Chicago world. But as we have not time or space to descant further upon this topic, we will leave it for some future communication, premising that we shall attack vigorously the hollowness of all such pretension; aye, even *ad deliquium animi.*

In one of our restless moods, the other night, we dropped into the Opera House to witness the performances of the Fox Pantomime Troupe, and it so forcibly recalled the younger days of our life,— alas, gone forever! — that, for a while, we were positively happy. The clown was excellent, and his gymnastics displayed so much ease, agility, and practiced skill, that each endeavor brought out rounds of applause. The Hungarian brothers, with their ballet dancers, came in every now and then, to fill up, and probably such very active and rapid movements were seldom witnessed. Their legs went through more contortions than a frog's, under the stimulus of the magnetic fluid; quickness, strength, etc., were the characteristics, but little grace; and how they got through, with such wretched music, is more than we can determine. We suppose, after so exemplary an evening, we were allowed to "crook our elbow," once, at least, and should not be d—d for such a lapse from virtue. But, oh! you pious members of our profession, don't turn up your eyes to Heaven, and pray the gory God, like the Pharisee of old, that you are not one of us. We know you from long experience, and know how many a square drink you take — behind the door, and how many iniquities you commit under the cover of your sanctity. It is much better, if you *will* drink, to do it openly, as a man; to do it in moderation; to throw the word policy, the synonym of hypocrisy, to the winds, and use, not abuse, every blessing vouchsafed to man; at the same time, admitting it a matter of taste with some, and education with others, as our old fathers always taught us to eschew sly tricks, and never to invade forbidden pastures. Farewell, oh! Professor; we shall meet soon again. More anon. "VIATOR."

CHICAGO, April, 1870.

